# Assessing effects of exoskeleton misalignment on knee joint load during swing using an instrumented leg simulator

**DOI:** 10.1186/s12984-022-00990-z

**Published:** 2022-01-29

**Authors:** Jule Bessler-Etten, Leendert Schaake, Gerdienke B. Prange-Lasonder, Jaap H. Buurke

**Affiliations:** 1 Roessingh Research and Development, Enschede, The Netherlands; 2 Department of Biomedical Signals and Systems, University of Twente, Enschede, The Netherlands; 3 Department of Biomechanical Engineering, University of Twente, Enschede, The Netherlands

**Keywords:** Exoskeletons, Joint misalignments, Rehabilitation, Safety, Joint load

## Abstract

**Background:**

Exoskeletons are working in parallel to the human body and can support human movement by exerting forces through cuffs or straps. They are prone to misalignments caused by simplified joint mechanics and incorrect fit or positioning. Those misalignments are a common safety concern as they can cause undesired interaction forces. However, the exact mechanisms and effects of misalignments on the joint load are not yet known. The aim of this study was therefore to investigate the influence of different directions and magnitudes of exoskeleton misalignment on the internal knee joint forces and torques of an artificial leg.

**Methods:**

An instrumented leg simulator was used to quantify the changes in knee joint load during the swing phase caused by misalignments of a passive knee brace being manually flexed. This was achieved by an experimenter pulling on a rope attached to the distal end of the knee brace to create a flexion torque. The extension was not actuated but achieved through the weight of the instrumented leg simulator. The investigated types of misalignments are a rotation of the brace around the vertical axis and a translation in anteroposterior as well as proximal/distal direction.

**Results:**

The amount of misalignment had a significant effect on several directions of knee joint load in the instrumented leg simulator. In general, load on the knee joint increased with increasing misalignment. Specifically, stronger rotational misalignment led to higher forces in mediolateral direction in the knee joint as well as higher ab-/adduction, flexion and internal/external rotation torques. Stronger anteroposterior translational misalignment led to higher mediolateral knee forces as well as higher abduction and flexion/extension torques. Stronger proximal/distal translational misalignment led to higher posterior and tension/compression forces.

**Conclusions:**

Misalignments of a lower leg exoskeleton can increase internal knee forces and torques during swing to a multiple of those experienced in a well-aligned situation. Despite only taking swing into account, this is supporting the need for carefully considering hazards associated with not only translational but also rotational misalignments during wearable robot development and use. Also, this warrants investigation of misalignment effects in stance, as a target of many exoskeleton applications.

## Background

Wearable robots for physical assistance and rehabilitation are playing an increasingly relevant role in our societies. The ageing population increases the need for technologies supporting people with chronic disability as well as the ageing workforce. Technical advancements enabling the use of robots in close interaction with humans across domains can help address this need [1]. Many different types of exoskeletons exist, along with a variety of application areas and use cases.

Especially in the field of rehabilitation robotics, misalignments are a common safety concern in exoskeleton use [2, 3]. A mismatch between the user’s anatomical joint and the exoskeleton joint can cause undesired interaction forces, which in turn can reduce comfort and safety [4, 5]. One can distinguish between two main causes of misalignments: a kinematic mismatch between the exoskeleton joint and the anatomical joint and wrong positioning or fitting of the exoskeleton. A kinematic mismatch between the exoskeleton joint and the anatomical joint is unavoidable as anatomical joints are too complex in their mechanics and kinematics to be perfectly mimicked by exoskeleton joints [6]. Limited degrees of freedom (DOF) in an exoskeleton can lead to misalignments during movement. To take the knee joint (as one of the less complex joints in the human body) as an example, its kinematics are characterized by a translation of the axis of rotation with increasing knee flexion [7]. This behavior is not replicated by the hinge joints that are typically used as an exoskeleton knee joint [5]. The micro-misalignments that are created in the course of a knee flexion (or the movement of any other joint supported by an exoskeleton) are therefore an inherent hazard. Wrong positioning or poor fit of an exoskeleton can lead to larger misalignments as the exoskeleton joint axis might be considerably translated or rotated with respect to the anatomical joint axis. As contemporary robotic exoskeleton systems, unlike many orthotic devices, are usually one-size-fits-all solutions, they can only be adjusted to the user to a certain degree by e.g., changing the segment length and adjusting straps that are used to attach the device to the user’s limbs. Careful utilization of those adjustment options by trained experts is vital to minimize misalignments. Inaccurate setting of the exoskeleton segment lengths can cause a proximal/distal translation of the exoskeleton joint axis with respect to the anatomical joint. Another source of poor positioning can be (potentially unsupervised) donning of the system by its user. The exoskeleton joint might be positioned in a way that its rotation axis is rotated with respect to the anatomical joint rotation axis. Moreover, deviations in soft tissue, clothing worn under the exoskeleton, cushion thickness or strap length could lead to the exoskeleton joint axis being translated (in anteroposterior direction) with respect to the anatomical joint axis. Misalignments can not only lead to undesired forces and torques on the musculoskeletal system but also to increased pressure and shear at the fixation points of the exoskeleton (e.g., cuffs), where a relatively too long or too short exoskeleton segment will push or pull on the user’s soft tissue [4, 7, 8]. Misalignments have previously been discussed as potential cause for bone fractures in lower limb exoskeleton use [9, 10].

Recent research efforts have focused on the development of misalignment compensation mechanisms [5, 6, 11]. Common strategies for misalignment compensation include manual alignment, the use of compliant elements, and the addition of kinematic redundancy. Manual alignment can reach good results but requires a trained person and can be time-consuming and is only a valid option if the exoskeleton joint’s mechanics are very similar to those of the anatomic joint. Compliant elements either at the frame and brace level or at the joint level can allow for small compensatory movements and thereby reduce the effects of small misalignments. Another option is to add kinematic redundancy, i.e., to add more DOF. This however often makes the device heavier and bulkier and can introduce additional inertia to the system [5]. Literature on the effects of misalignments is scarce. Some studies investigated the effects of compensation mechanisms on forces at the skin-cuff interface [12, 13] and one study introduced a misalignment to a lower limb exoskeleton to study the effects on gait [8]. Due to the limited amount of studies, there is an overall lack of knowledge regarding misalignments in wearable robots. This includes missing information on the occurrence of adverse events caused by misalignments, effects of misalignments on comfort and safety of exoskeleton users, and acceptable levels of misalignment. Assuming that misalignments cannot be prevented completely, knowledge on acceptable levels of misalignment not causing significant harm to the exoskeleton user is required. As a first step towards this knowledge, the mechanisms behind misalignments and its influences on forces acting on the body need to be better understood.

The aim of this study is to investigate the influence of different directions and magnitudes of misalignments on the forces and torques in the musculoskeletal system using a dummy limb. The approach of using a dummy limb with simple mechanics and readily available components was chosen with the exoskeleton developer in mind who is regularly confronted with the challenge of testing and validating device safety. In addition to the use in the present study, it can be a first step into developing a system that can be replicated by others that want to assess effects of misalignments in their device. We hypothesized that the forces and torques applied to the knee joint during exoskeleton use are higher when a misalignment is introduced compared with the well-aligned situation. We investigate the effects of different directions of misalignment between the knee joint of a passive leg simulator and the joint of a passive knee brace being manually flexed. The investigated directions of misalignment include a rotation around the vertical axis and a translation in anteroposterior as well as proximal/distal direction.

## Methods

A series of experiments was executed using an instrumented leg simulator (ILS) and a passive knee brace in different alignment settings, which was manually flexed by the experimenter. The apparatus and procedures are explained in detail in the following sections.

### Apparatus

**Fig. 1:**
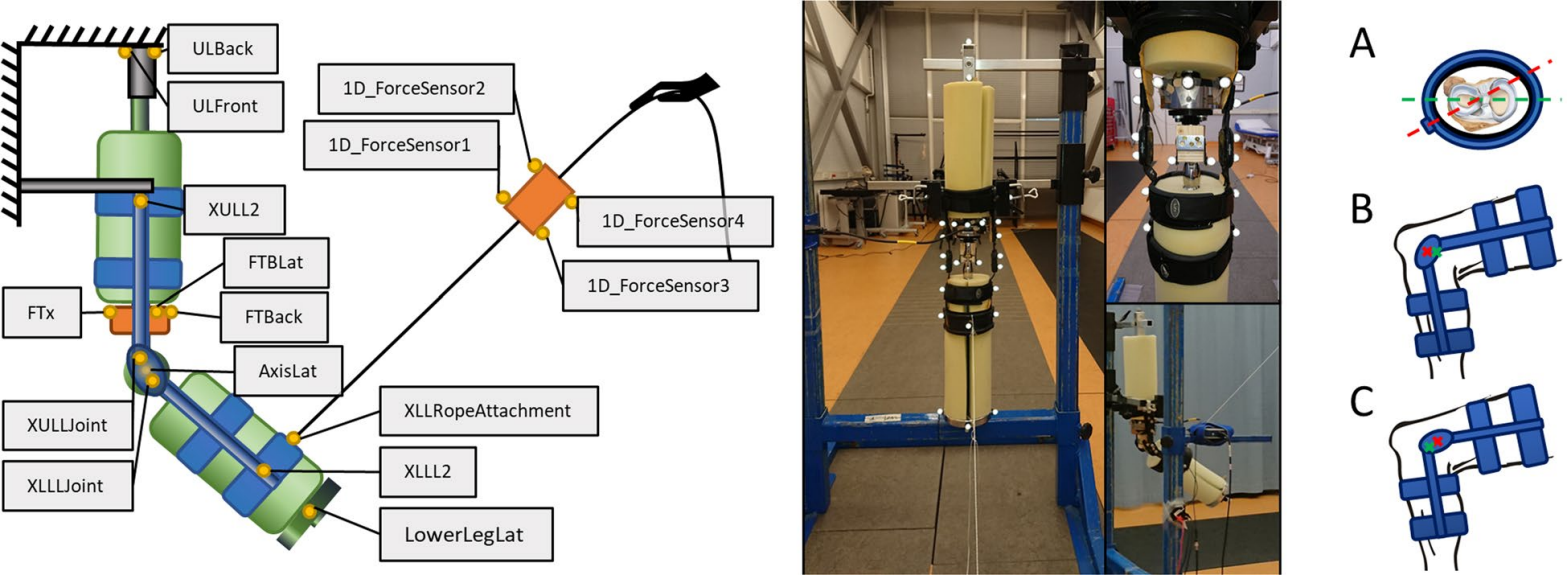
Overview of the setup. Left: Schematic representation of the setup and marker placement, lateral view. The ILS is shown in green, the orthosis in blue, the frame in grey/black, the sensors in orange and the markers in yellow. Markers XULM2, FTFM, AxisMed, XULMJoint, XLLMJoint, XLLM2 and LowerLegMed are not shown as they are placed on the medial side. Detailed information about marker placement can be found in the Annex. Middle: Photos of the setup. Right: Visualization of internal rotational misalignment (**A**), posterior translational misalignment (**B**) and distal translational misalignment (**C**) of a leg with respect to a knee brace, where the red dashed line and crosses represent the orthosis center of rotation and the green dashed line and crosses represent the leg center of rotation

The ILS has been specifically developed and built for this study. It consists of two rigid segments which are connected through a simple hinge joint and covered with a compliant material to mimic soft tissue (Fig. 1). The rigid part of the leg segments mimicking the bones consists of two aluminum tubes of 22 mm diameter and 2 mm wall thickness. The upper and lower segment are connected through a simple hinge joint with a centered rotation axis (Beukenholdt zeilmakerij, Leiderdorp, the Netherlands). To measure the effects of misalignments with regard to forces and torques on the musculoskeletal system, the ILS is instrumented using a 6 DOF force and torque (FT) sensor (ATI FT-Delta DI60-660, ATI Industrial Automation, Apex, North Carolina, USA), which is connected to the bottom of the upper rod and the top of the joint using custom 3D-printed parts. The soft tissue is mimicked by a hollow polyether foam rubber cylinder with a specific gravity (SG) of 40, an inner diameter of 25 mm and an outer diameter of 130 mm (Joan’s Comfortschuim BV, IJmuiden, the Netherlands). A circular weight is attached to the distal end of the lower leg segment. The total weight of the ILS is 4.2 kg and the lower leg weight is 3.3 kg.

To mimic leg exoskeleton use in a simple way, we used a passive semi-rigid knee brace (A. C. lite by DonJoy, DJO, LLC, Lewisville, Texas, USA) with a polycentric joint and straps above and below the knee, which is commonly used to support knee ligament instabilities. The brace can be attached to the ILS and then moved by the experimenter by pulling on a string attached to the distal posterior strap of the orthosis (see Fig. 1). To quantify the pulling force that generates the flexion torque at the orthosis joint, a tensile force sensor (Model No. 615, Tedea-Huntleigh, Chatsworth, California, USA) is attached to the string. The ILS is symmetrical and therefore does not have specific characteristics of a left or right leg. However, the used knee brace is designed for the left leg and therefore the ILS will be considered as a left leg simulator in this article. The upper orthosis segment was mounted in a steel frame to keep it in place. To avoid slipping of the ILS due to its weight, its upper end was also fixed to the same frame.

### Procedure

At the start of the experiment, the ILS and brace were aligned as good as possible by an experienced researcher based on visual assessment. They were positioned inside the measuring volume of an 8 camera marker-based optoelectronic tracking system (Vicon Nexus with 4 Vero and 4 Vantage cameras, VICON, Oxford UK). The apparatus was equipped with 33 markers (Fig. 1; details about marker placement can be found in the Annex) to define its segments and track their movement. The tensile force sensor was connected to an analog input channel of the optoelectronic measurement system and the 6 DOF FT sensor was connected to a separate PC to record the data via in-house developed software. A synchronization signal was captured by both measurement systems to enable offline synchronization.

The alignment settings were achieved by manually moving the ILS with respect to the orthosis or rotating its joint in the setup. We attempted to reach different amounts of misalignment by visual inspection. The alignments were modified independently such that when one direction of misalignment was modified, the other two directions were kept at perfect alignment as far as that was possible based on visual inspection. The exact alignment parameters were later calculated using the marker data, to be used for analysis. For each of the alignment settings, at least two trials were recorded with each trial containing at least 10 knee flexions. The 10 knee flexions were exerted by the experimenter pulling on the string attached to the distal end of the orthosis. The extensions were not actuated but achieved through the weight of the distal ILS segment. The three directions of misalignment were:

- Rotational misalignment: A rotation of the knee flexion axis with respect to the orthosis flexion axis around the vertical (z-) axis. An alignment angle of zero represents a perfect rotational alignment of the two flexion axes, negative values correspond to an internal rotation of the ILS knee joint with respect to the knee brace joint and positive values correspond to an external rotation of the ILS knee joint with respect to the knee brace joint. We aimed for misalignments in a range of ± 20 deg in steps of about 5 deg. From the ILS/patient perspective, negative rotational misalignment values would correspond to an external rotation and positive values to an internal rotation of the brace.
- Translational misalignment in anteroposterior direction: A horizontal displacement of the ILS joint (in x-direction). A negative alignment distance represents a posterior translation of the ILS joint with respect to the knee brace joint and a positive alignment distance represents an anterior translation of the ILS joint axis with respect to the knee brace axis. We aimed for misalignments in a range of ± 10 mm in steps of 10 mm. From the ILS/patient perspective, negative anteroposterior misalignment values would correspond to anterior and positive values to a posterior translation of the brace.
- Translational misalignment in proximal/distal direction: A vertical displacement of the ILS (in z-direction). Negative values correspond to the ILS joint being translated distally with respect to the knee brace axis and positive values correspond to the ILS joint being translated proximally with respect to the knee brace axis. We aimed for misalignments in a range of ± 20 mm in steps of 10 mm. From the ILS/patient perspective, negative proximal/distal misalignment values would correspond to proximal and positive values to a distal translation of the brace.

For consistency, from this point onward, when using the terms internal/external rotation, anterior/posterior translation and proximal/distal translation, we refer to the device perspective. Thus, an internal rotation means that the ILS knee is rotated inwards with respect to the brace joint, an anterior translation means that the ILS knee is forward translated with respect to the brace joint, and a proximal translation means that the ILS knee is located higher than the brace joint.

We repeated the rotational misalignment trials in two brace strap pressure settings to investigate whether this has an effect on the outcomes in measured forces and torques. As we were not able to measure strap pressure in the current setup, we could only group by “tight straps” and “loosened straps”. In the “tight straps” setting, the straps of the lower orthosis segment were pulled tight so that the foam was compressed while in the “loosened straps” setting the straps were closed without visibly compressing the foam with fully extended ILS. This additional condition was only tested in the rotational misalignment experiments since contrary to translational misalignments, an adjustment of the rotational misalignment did not require a re-attachment of the straps. Therefore, the two distinct settings were only applicable in the rotational misalignment session. The upper orthosis straps remained at approximately the same strap pressure during the entire experiment. For calibration, two static trials were recorded in which the lower leg of the ILS was hanging freely without the orthosis attached and without any movements being executed.

### Data processing and analysis

The marker data were processed and labelled in Vicon Nexus 2.9.2 using a custom kinematic model and then exported for further analysis in Python 3.7.4. All data (marker data, FT sensor data, tensile force sensor data) were filtered using a low-pass bidirectional Butterworth filter of 4th order with a cutoff frequency of 6 Hz. We used the segment orientations to calculate the ILS and orthosis flexion angles as well as the rotational alignment as defined above. We did this by computing the rotation matrices of the lower segments in the local coordinate systems of the upper segments and the rotation matrices of the ILS segments in the local coordinate systems of the orthosis segments respectively and computing the Euler angles. The translational alignment in x- and z-direction (i.e., anteroposterior and proximal/distal alignment) was calculated as the distance of the ILS knee joint center (midpoint of markers AxisMed and AxisLat) from the orthosis joint center (midpoint of markers XULMJoint, XULLJoint, XLLMJoint and XLLLJoint). To extract one single alignment value per trial and misalignment type, we took the mean of the considered alignment parameter in the static situation before the first knee flexion (i.e., from the beginning of the trial to the point where the ILS flexion angle increases to more than 120% of the initial ILS flexion angle).

**Fig. 2:**
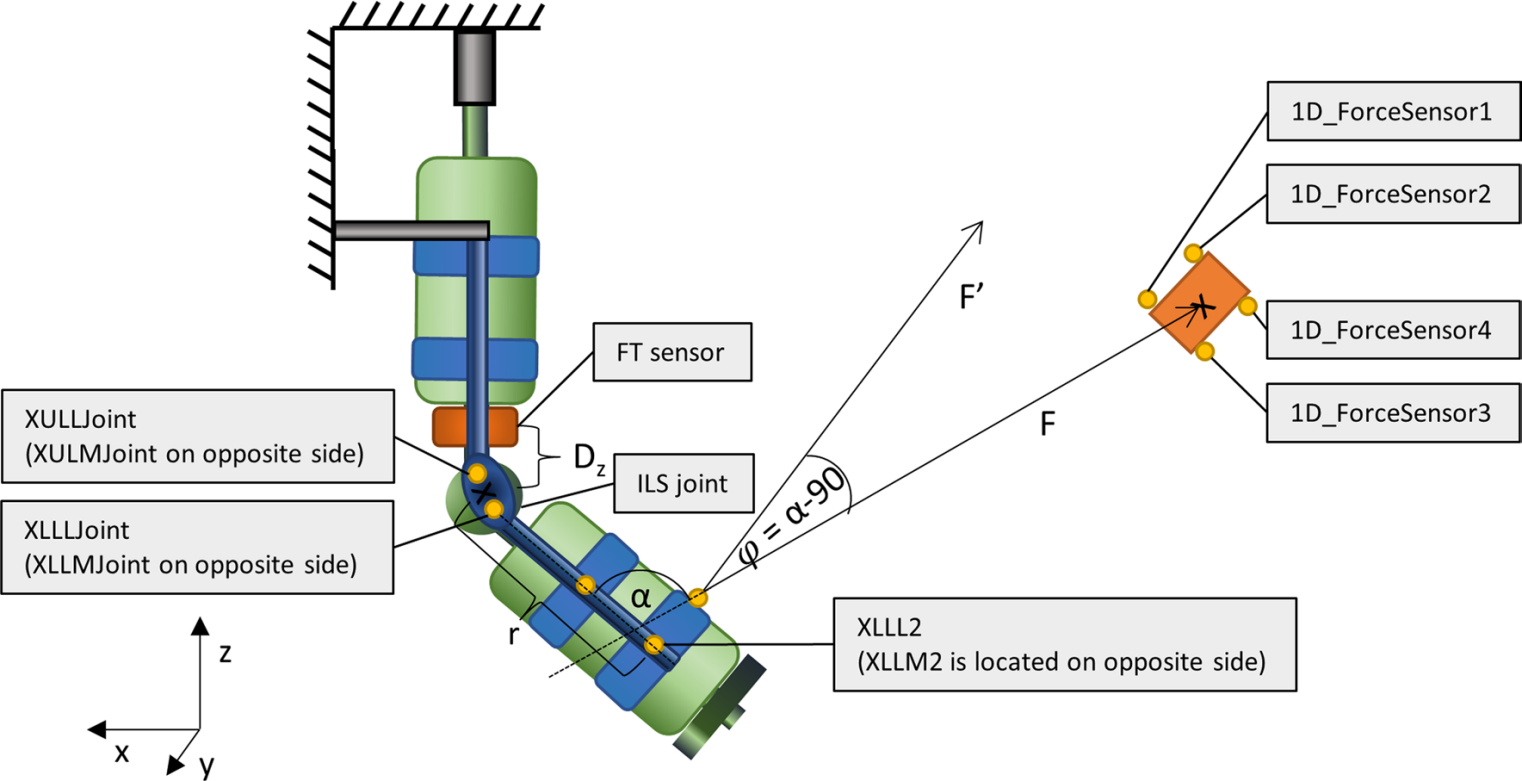
Schematic representation of the setup and measures used for calculating the forces and torques at the joint location as well as the actuation torque created by the pulling force

FT sensor data were corrected by subtracting the offset values obtained from the calibration recordings. The offset values were achieved by calculating the means of the FT sensor recordings from one of the calibration trials and then subtracting the lower leg weight from the Fz value. The offset values were subtracted from the FT sensor data for the entire time series of each trial before any further processing. The forces and torques at the ILS joint location were calculated from the recorded forces and torques at FT sensor location and the distance between the FT sensor reference frame origin and the ILS joint sensor. With the assumption that the part of the setup between the ILS joint and the FT sensor is rigid, we used the mean of the vertical distance (z-direction) in the static situation before the first flexion, similarly to the alignment calculations. We further assumed that the distance in x and y direction was zero due to the ILS design. This resulted in the following formulas:

$$\begin{aligned}&{F{x}_{joint}=F{x}_{sensor}+ \frac{M{y}_{sensor}}{Dz}}\\ &{F{y}_{joint}=F{y}_{sensor}- \frac{M{x}_{sensor}}{Dz}}\\ &{F{z}_{joint}=F{z}_{sensor}}\end{aligned}$$

$$\begin{aligned}&{M{x}_{joint}=M{x}_{sensor}+Dz F{y}_{sensor}} \\ &{M{y}_{joint}=M{y}_{sensor}- Dz F{x}_{sensor}} \\ &{M{z}_{joint}=M{z}_{sensor}}\end{aligned}$$

where $F{x}_{sensor}$, $F{y}_{sensor}$ and $F{z}_{sensor}$ are the forces measured by the FT sensor, $M{x}_{sensor}$, $M{y}_{sensor}$ and $M{z}_{sensor}$ are the torques measured by the FT sensor and $Dz$ is the vertical distance between the ILS joint center and FT sensor reference frame origin. $Dz$ is negative as the ILS joint center is located below the FT sensor (Fig. 2). For readability, $F{x}_{joint}$, $F{y}_{joint}$ and $F{z}_{joint}$ will be referred to as Fx, Fy and Fz and $M{x}_{joint}$, $M{y}_{joint}$ and $M{z}_{joint}$ will be referred to as Mx, My and Mz.

The flexion torque at the orthosis joint applied by the experimenter pulling on the string was calculated as follows:

$$\tau =r\,\mathrm{cos}\,\varphi\, F$$

where $r$ is the distance between the orthosis joint center (midpoint of markers XULLJoint, XULMJoint, XLLLJoint and XLLMJoint) and the origin of the lower orthosis segment (midpoint of markers XLLL2 and XLLM2), *F* is the force measured by the tensile force sensor and $\varphi$ is the angle between the actual tensile force vector and the force vector *F’* acting perpendicularly to the lower orthosis segment. It is calculated as the angle between the z-axis vector of the lower orthosis segment and the force vector of the tensile force (i.e., vector from XLLRopeAttachment marker to midpoint between all four 1D_ForceSensor markers) projected onto the x–z-plane, minus 90 degrees (Fig. 2).

For comparison between trials, we extracted peak forces (Fx_peak_, Fy_peak_, Fz_peak_) and torques (Mx_peak_, My_peak_, Mz_peak_) per repetition of the flexion/extension cycle for each of the trials. This was achieved by detecting the peaks in flexion torque, then slicing the data halfway between those peaks and finding the index of maximum absolute deviation from the starting value per force/torque component and slice. The ILS joint forces and torques at those indices were then extracted as peak forces/torques per repetition and trial. The means and standard deviations of all peaks per trial were later calculated to provide a better overview. Furthermore, peak flexion torques at the orthosis joint and peak ILS flexion angles per repetition were extracted.

The relationships between the following parameters were analyzed statistically employing a regression analysis:

- Misalignments and peak forces/torques at the ILS joint
- peak ILS flexion angle and misalignments
- Misalignments and peak manually applied flexion torque

For the analysis of misalignments and peak forces/torques at the ILS joint, we performed both a regression analysis of only absolute values to assess relationships between absolute misalignment and absolute joint load, and a regression analysis of the original values to also take the direction of misalignment as well as forces/torques into consideration. Regression lines with R-squared values larger than 0.7 are reported and considered as strong relationships. If no linear regression reaching this target was found, a 2nd degree polynomial regression analysis was performed with the same target R-squared. We employed a significance level of 0.05 for the probability of the F-statistic.

## Results

We recorded and analyzed 22 rotational misalignment trials ranging from 13 deg internal rotation to 19 deg external rotation, 6 anteroposterior translational misalignment trials ranging from 4 mm posterior to 14 mm anterior translation of the ILS joint center and 12 proximal/distal translational misalignment trials ranging from 23 mm distal translation to 12 mm proximal translation of the ILS joint center. Please note that two aligned trials were used in both the rotational misalignment and anteroposterior translational misalignment analysis (marked in Tables 2 and 4).

**Fig. 3:**
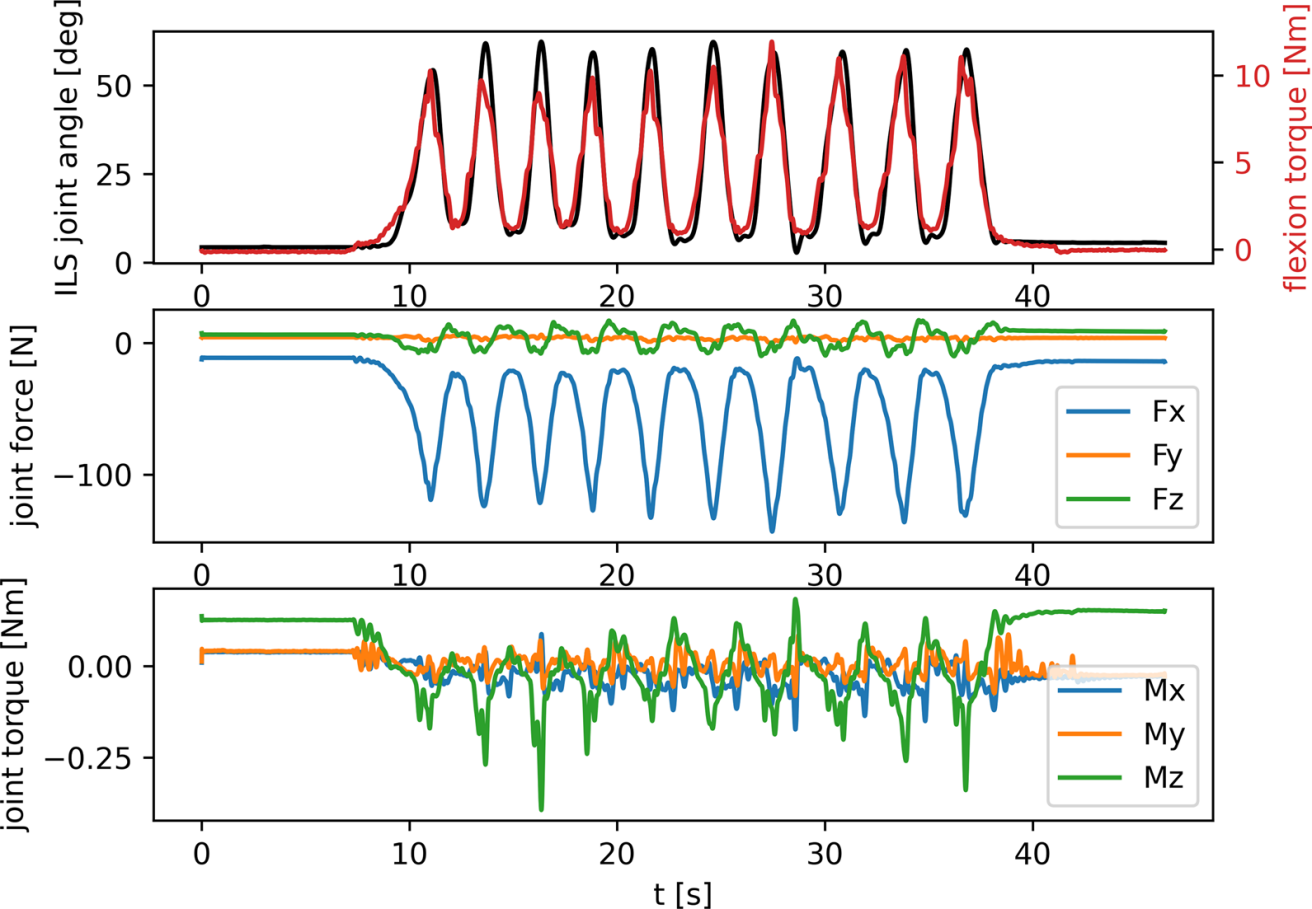
ILS joint angle, flexion torque and joint forces and torques over the course of a typical trial in aligned setup. The top graph shows the flexion angle of the ILS where 0 deg is full extension (black) and the flexion torque generated through the pulling force applied to the orthosis (red). The middle graph shows the forces in the ILS joint where positive Fx is forward directed force, positive Fy is pointing from medial to lateral, and positive Fz is compressive force. The bottom graph shows the torques in the ILS joint where positive Mx is adduction torque, positive My is flexion torque, and positive Mz is external rotation torque. See also Fig. 2 for axis orientation

Figure 3 shows a typical trial in an aligned setting, in which the ten repetitions of flexion and extension are clearly visible from the flexion torque and ILS joint angle data. As expected, while all torques as well as the forces in y- and z-direction stay close to zero throughout the trial, there is a clear increase of force in negative x-direction (i.e., the direction of the pulling force) with each ILS flexion.

### Effects of misalignments on joint forces and torques

The results of the effects of all three investigated directions of misalignments are presented in the following sections.

#### Rotational misalignment

**Fig. 4:**
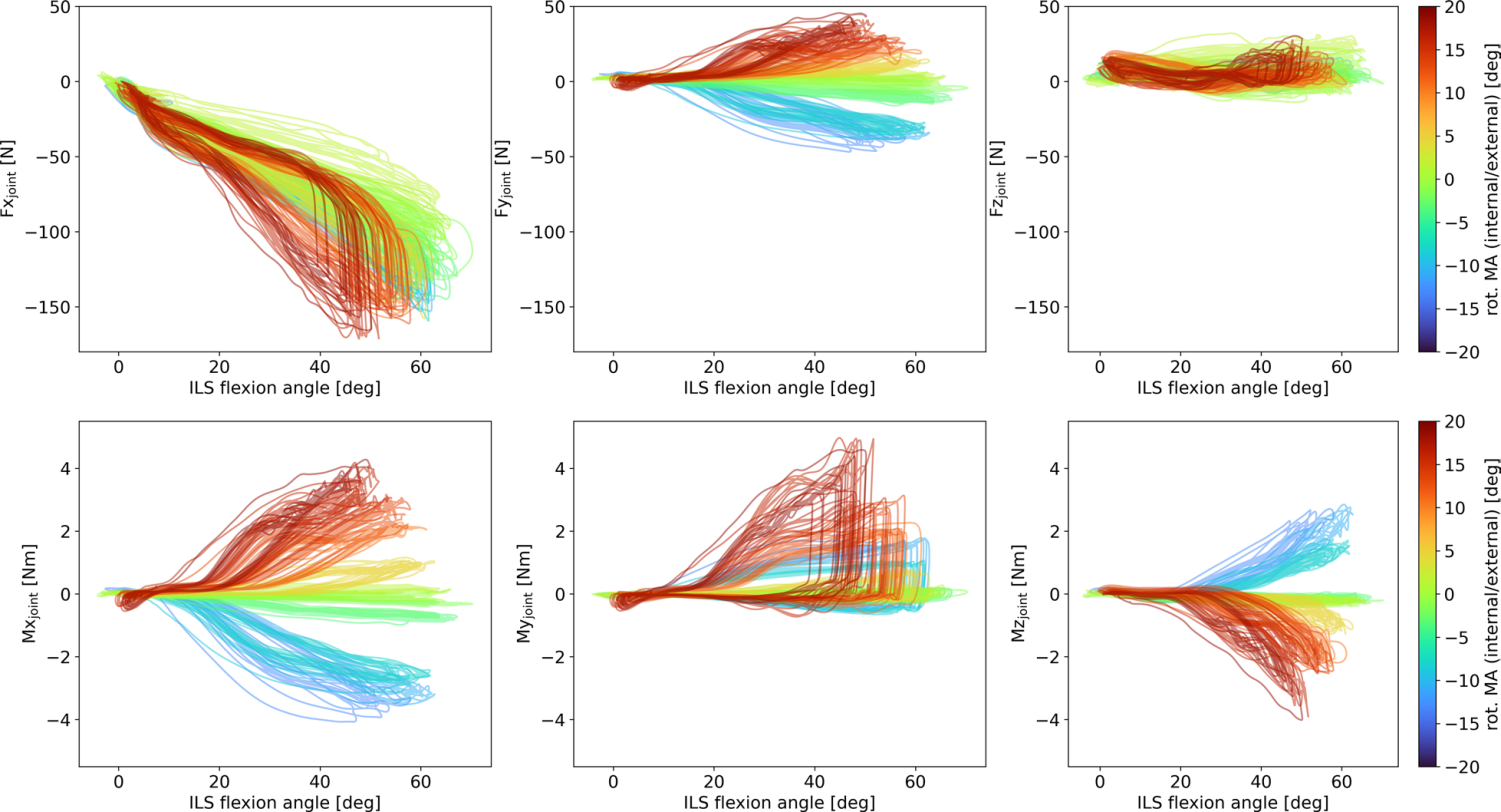
Hysteresis plots of ILS joint forces and torques over the ILS flexion angle. The amounts of rotational misalignment (rot. MA) are represented by the graph colors with darker blue shades representing stronger internal rotation and darker red shades representing stronger external rotation

With a larger degree of rotational misalignment, higher absolute peak forces and torques were observed for Fy, Mx, My and Mz, when only looking at amount of misalignment, regardless of the direction of misalignment (Table 1). When considering a more detailed picture including direction of misalignment, we can also see that rotational misalignment clearly has an effect on joint forces in x- and y-direction (Fig. 4). The peaks in negative x-direction around maximum flexion increase in magnitude with increasing rotational misalignments in both directions. A hysteresis is visible with a close to linear buildup of the force which is then released quickly with a large part of the force reduction happening before ILS joint extension. Joint force in y-direction is increasing with increasing joint angle when there is an external rotation misalignment and decreasing with increasing joint angle when there is an internal rotation misalignment. As mentioned above, the absolute peaks increase with increasing amount of misalignment in both directions. The pattern of joint force in z-direction varies per trial and the peaks do not show a clear relation with misalignment.

**Table 1:** Results of regression analysis for absolute values of misalignment and joint forces/torques

Misalignment	|Fx_peak_|	|Fy_peak_|	|Fz_peak_|	|Mx_peak_|	|My_peak_|	|Mz_peak_|
rotational	–	y = 2.20x + 3.82; R^2^ = 0.84	–	y = 0.23x + 0.08; R^2^ = 0.92	y = 0.20x − 0.17; R^2^ = 0.86	y = 0.16x + 0.12; R^2^ = 0.89
Translational anteroposterior	y = − 0.19x^2^ + 6.46x + 97.52; R^2^ = 0.70	–	–	y = 0.06x + 0.08; R^2^ = 0.94	–	–
Translational proximal/distal	–	–	–	–	–	–

In the regression equations, x is the absolute amount of misalignment (in degrees for rotational misalignment and in mm for translational misalignments) and y is the outcome measure as indicated in the top row in N or Nm respectively. A dash indicates that first and second degree polynomial fits did not meet the criterium of R^2^ > 0.7. All p-values were p < 0.01

Joint torques around the x-axis are increasing in positive direction with increasing joint angle when there is an external rotation misalignment and increasing in negative direction with increasing joint angle when there is an internal rotation misalignment. Joint torques around the z-axis show a similar pattern in the opposite direction. Torques around the y-axis have peaks at around maximum flexion that strongly increase with increasing amount of misalignment in both directions. Trials with larger misalignments show a steeper increase in joint forces (except in z-direction) and torques with increasing flexion angle. Especially the trials with large misalignments show a very strong hysteresis; after a steady buildup of force or torque, there is a sudden drop at maximum flexion.

The means and standard deviations of the peak forces and torques per flexion/extension cycle and the amount of rotational misalignment per trial are shown in Table 2. Similar to the outcomes regarding the absolute misalignment values, the analysis concerning directions of misalignment revealed a positive linear regression between the amount of rotational misalignment and peak Fy ($F{y}_{\mathrm{peak}}=2.61 Alignmen{t}_{rot}-3.63; {R}^{2}=0.95; p<0.01$) as well as peak Mx ($M{x}_{peak}=0.24 Alignmen{t}_{rot}-0.26; {R}^{2}=0.98; p<0.01$). For the relationship between the amount of rotational misalignment and peak My, we found a 2nd degree polynomial fit ($M{y}_{peak}=0.01 Alignmen{t}_{rot}^{2}+0.02 Alignmen{t}_{rot}; {R}^{2}=0.90;p<0.01$) and for the relationship between the amount of rotational misalignment and peak Mz, we found a negative linear regression $\left( {Mz_{peak} = - 0.16Alignment_{rot} - 0.18;\,R^{2} = 0.92;\,p < 0.01} \right)$.

**Table 2:** Peak forces and torques presented as M (SD) per rotational misalignment trial

Rotational alignment [deg]	Fx_peak_ [N]	Fy_peak_ [N]	Fz_peak_ [N]	Mx_peak_ [Nm]	My_peak_ [Nm]	Mz_peak_ [Nm]
− 12.9	− 127.3 (8.2)	− 41.6 (3.0)	6.5 (8.5)	− 3.63 (0.21)	1.58 (0.09)	2.37 (0.22)
− 10.7	− 143.5 (7.5)	− 36.9 (1.9)	14.0 (1.8)	− 3.32 (0.12)	1.61 (0.13)	2.48 (0.24)
− 9.0*	− 140.1 (6.7)	− 34.4 (2.4)	− 3.1 (2.2)	− 2.83 (0.15)	1.01 (0.03)	1.51 (0.16)
− 8.1*	− 140.9 (9.2)	− 33.1 (1.8)	1.2 (6.9)	− 2.68 (0.06)	0.97 (0.04)	1.47 (0.13)
− 3.5*	− 127.2 (9.2)	− 12.3 (2.1)	− 1.2 (1.0)	− 0.83 (0.07)	− 0.29 (0.02)	− 0.23 (0.02)
− 3.1*	− 126.7 (6.0)	− 12.7 (1.5)	4.0 (8.5)	− 0.74 (0.02)	− 0.27 (0.01)	− 0.23 (0.03)
− 1.8 ~	− 119.6 (5.2)	− 7.7 (0.7)	6.5 (6.7)	− 0.34 (0.03)	− 0.21 (0.01)	− 0.26 (0.02)
− 1.6 ~	− 113.4 (7.6)	− 5.9 (0.6)	10.8 (0.7)	− 0.32 (0.04)	− 0.20 (0.01)	− 0.27 (0.04)
− 1.0	− 108.7 (4.7)	− 2.9 (1.3)	9.2 (1.5)	− 0.20 (0.02)	− 0.19 (0.01)	− 0.30 (0.04)
− 0.5*	− 129.8 (6.8)	1.5 (1.7)	− 8.5 (1.2)	− 0.11 (0.03)	− 0.06 (0.01)	− 0.24 (0.08)
− 0.1	− 98.7 (3.7)	− 1.9 (0.7)	12.8 (1.5)	− 0.22 (0.02)	− 0.17 (0.02)	− 0.36 (0.05)
− 0.1*	− 96.7 (5.5)	0.1 (0.8)	19.5 (1.6)	− 0.01 (0.06)	− 0.10 (0.14)	− 0.24 (0.03)
0.3*	− 120.9 (22.8)	2.9 (3.2)	− 3.5 (11.6)	− 0.09 (0.07)	0.06 (0.05)	− 0.21 (0.15)
1.8*	− 77.1 (12.1)	7.0 (1.0)	27.5 (3.1)	0.20 (0.12)	0.29 (0.03)	− 0.29 (0.05)
4.3*	− 127.8 (5.7)	14.8 (2.2)	− 6.1 (1.4)	1.00 (0.08)	0.64 (0.07)	− 1.00 (0.17)
4.8*	− 129.9 (12.3)	13.7 (1.4)	− 5.3 (0.9)	0.97 (0.06)	0.66 (0.08)	− 1.00 (0.17)
10.2*	− 148.6 (4.9)	21.4 (1.2)	− 5.2 (2.2)	2.17 (0.05)	2.01 (0.11)	− 2.08 (0.16)
12.6*	− 147.6 (4.1)	27.5 (3.3)	− 4.4 (1.2)	2.41 (0.22)	2.00 (0.07)	− 2.24 (0.22)
13.2	− 149.9 (5.5)	27.0 (1.7)	17.5 (1.7)	2.95 (0.11)	2.77 (0.16)	− 2.22 (0.19)
16.1*	− 167.5 (4.5)	38.5 (2.5)	22.4 (3.5)	3.78 (0.22)	4.54 (0.41)	− 3.24 (0.32)
16.2	− 148.6 (4.5)	32.8 (2.9)	14.3 (1.9)	3.21 (0.16)	2.94 (0.17)	− 2.22 (0.20)
18.6*	− 143.3 (12.8)	38.4 (3.3)	6.7 (13.5)	3.77 (0.27)	3.67 (0.51)	− 2.68 (0.56)

Trials marked with * in the first column were the trials with increased strap pressure. Trials marked with ~ in the first column were also used in the anteroposterior translational misalignment analysis

#### Effects of strap pressure

In the present data, the different levels of strap pressure did not have an effect on the ILS joint peak forces and torques. The regression for the pooled data (tight and loose straps) explained at least the same proportion of the variance as the least performing regression of the two data subsets (Table 3).

**Table 3:** Results of regression analysis for tight straps, loosened straps and both conditions pooled

Condition	Fy_peak_	Mx_peak_	My_peak_	Mz_peak_
tight straps	y = 2.61x − 2.99; R^2^ = 0.94	y = 0.24x − 0.25; R^2^ = 0.98	y = 0.01x^2^ + 0.03x + 0.12; R^2^ = 0.89	y = − 0.16x − 0.26; R^2^ = 0.92
loosened straps	y = 2.56x − 5.03; R^2^ = 0.97	y = 0.24x − 0.27; R^2^ = 0.98	y = 0.01x^2^ + 0.01x − 0.11; R^2^ = 0.94	y = − 0.17x − 0.07; R^2^ = 0.91
pooled (both)	y = 2.61x − 3.73; R^2^ = 0.95	y = 0.24x − 0.26; R^2^ = 0.98	y = 0.01x^2^ + 0.02x + 0.05; R^2^ = 0.90	y = − 0.16x − 0.18; R^2^ = 0.92

In the regression equations, x is the amount of rotational misalignment in degrees and y is the outcome measure as indicated in the top row in N or Nm respectively. All p-values were p < 0.01

#### Translational misalignment in anteroposterior direction

**Fig. 5:**
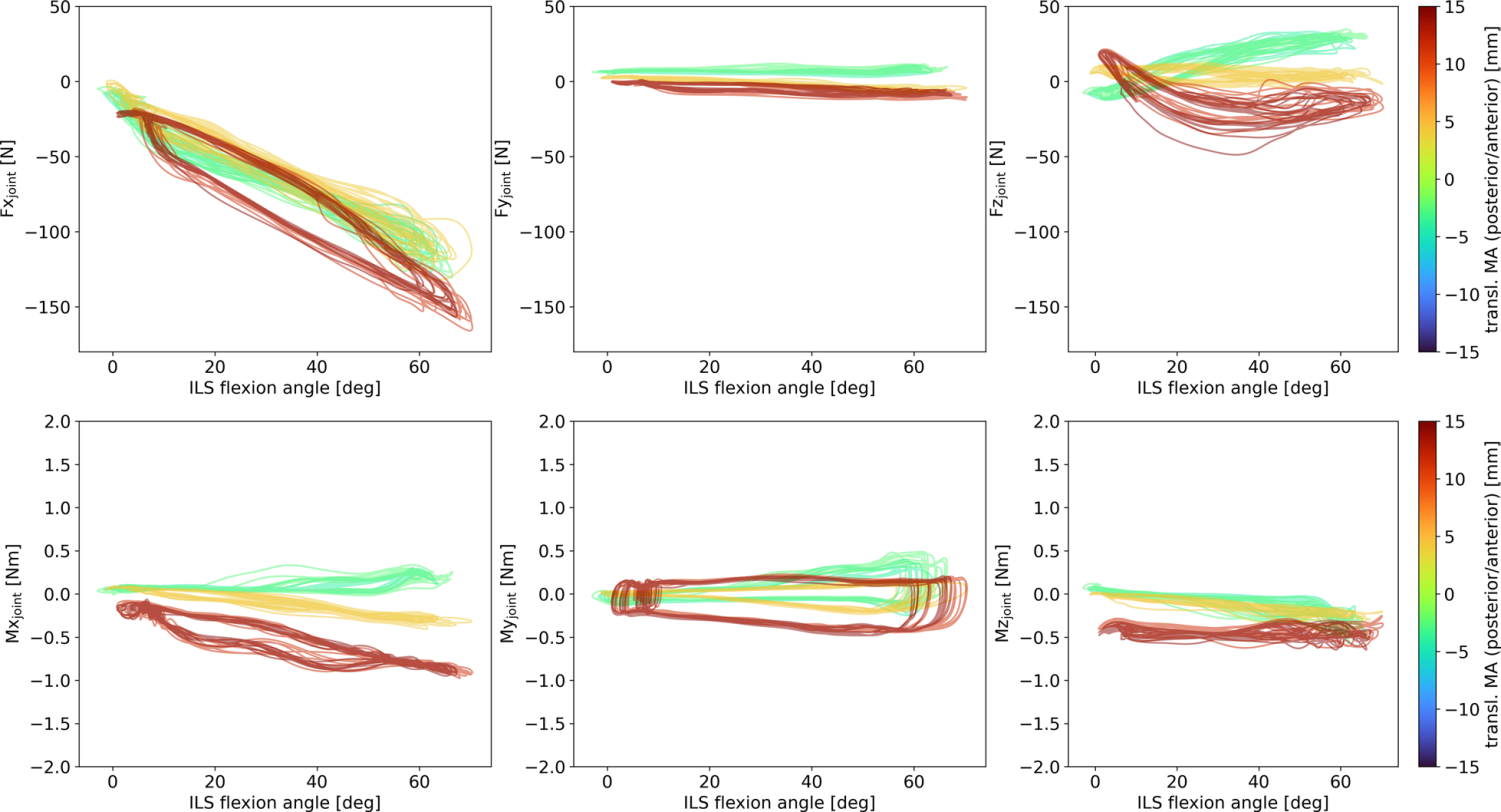
Hysteresis plots of ILS joint forces and torques over the ILS flexion angle. The amounts of anteroposterior translational misalignment (transl. MA) are represented by the graph colors with green–blue shades representing posterior translation and orange-red shades representing anterior translation

As the current setup does not allow for easy translation of the orthosis in anteroposterior direction, only three different levels of anteroposterior alignment could be achieved (see Table 4, note that each misalignment setting was measured twice and there are slight variations in measured amount of misalignment in those two repetitions per setting). With a larger degree of anteroposterior misalignment, higher absolute peak forces and torques were observed for Fx (2nd degree polynomial fit) and Mx (linear fit), when only looking at amount of misalignment, regardless of the direction of misalignment (Table 1). When considering a more detailed picture including direction of misalignment, this pattern is confirmed in Fx, showing a higher absolute peak force and stronger hysteresis with larger misalignment (Fig. 5). Fy is low overall, stays almost constant across the flexion/extension circle and varies slightly in starting values in the different conditions. The starting values of Fz are influenced by the misalignment in an opposite manner than those of Fy. The shapes of the curves clearly differ in the different alignment settings showing a positive peak at maximum flexion in the posterior translation setting and a positive peak at maximum extension and a negative peak at around half of the maximum flexion in the anterior translation setting. Mx shows only little variation in the aligned and posterior translation settings and a decrease with increasing flexion in the anterior translation setting. A tendency for positive peaks in posterior translation misalignment and negative peaks in anterior translation misalignment is visible. My and Mz show comparable behavior in all settings covering a small torque range.

**Table 4:** Peak forces and torques presented as M (SD) per anteroposterior translational misalignment trial

Translational alignment anteroposterior [mm]	Fx_peak_ [N]	Fy_peak_ [N]	Fz_peak_ [N]	Mx_peak_ [Nm]	My_peak_ [Nm]	Mz_peak_ [Nm]
− 4.0	− 113.5 (4.9)	7.1 (3.0)	31.1 (1.2)	0.27 (0.02)	0.33 (0.04)	− 0.33 (0.09)
− 2.5	− 123.8 (4.3)	10.2 (1.1)	32.4 (1.1)	0.27 (0.04)	0.43 (0.04)	− 0.45 (0.07)
3.9 ~	− 113.4 (7.6)	− 5.9 (0.6)	10.8 (0.7)	− 0.32 (0.04)	− 0.20 (0.01)	− 0.27 (0.04)
4.3 ~	− 119.6 (5.2)	− 7.7 (0.7)	6.5 (6.7)	− 0.34 (0.03)	− 0.21 (0.01)	− 0.26 (0.02)
11.5	− 155.2 (6.2)	− 10.5 (1.1)	− 27.6 (2.7)	− 0.91 (0.03)	− 0.44 (0.01)	− 0.41 (0.15)
14.4	− 145.4 (6.4)	− 9.0 (0.9)	6.4 (24.9)	− 0.90 (0.04)	− 0.44 (0.03)	− 0.38 (0.09)

Trials marked with ~ in the first column were also used in the rotational misalignment analysis

Regression analysis of the peak forces and torques (Table 4) resulted in a negative linear relationship between anteroposterior translation and peak Fy ($F{y}_{peak}=-1.07 Alignmen{t}_{transAP}+2.27;{R}^{2}=0.75; p<0.01$), peak Mx ($M{x}_{peak}=-0.07 Alignmen{t}_{transAP}+0.01; {R}^{2}=0.97; p<0.01$) as well as peak My ($M{y}_{peak}=-0.05 Alignmen{t}_{transAP}+0.13; {R}^{2}=0.88; p<0.01$).

#### Translational misalignment in proximal/distal direction

**Fig. 6:**
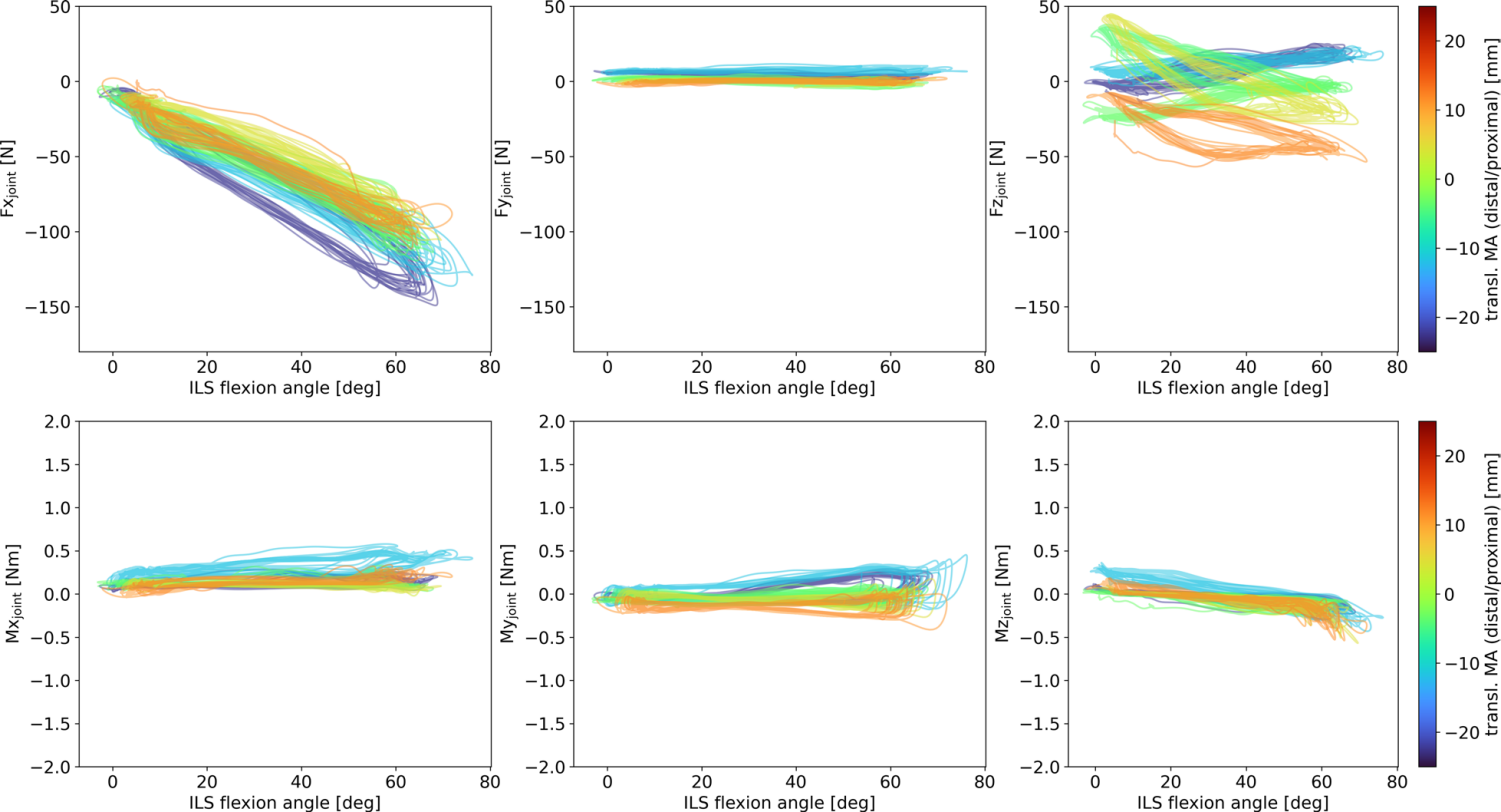
Hysteresis plots of ILS joint forces and torques over the ILS flexion angle. The amounts of proximal/distal translational misalignment (transl. MA) are represented by the graph colors with green–blue shades representing distal translation and orange–red shades representing proximal translation

For proximal/distal translational misalignment, there were no relationships (with R^2^ > 0.7) between amount of misalignment and absolute peak joint forces or torques, when only looking at amount of misalignment, regardless of the direction of misalignment (Table 1). When looking at the ILS joint forces and torques over the flexion/extension cycle in all proximal/distal translational misalignment settings (Fig. 6), a more nuanced picture is observed. Fx shows an increase of absolute peak force and hysteresis with larger misalignments, while Fy shows little change over the flexion/extension cycle but a slight variance in starting values. The starting values and shapes of Fz curves vary per trial with a tendency of more pronounced negative peaks and hysteresis in the proximal translation trials. Joint torques show little variance among the trials, except for the two trials at approximately 12 mm distal translation showing higher positive peaks in all directions, in y-direction together with the trials in strongest distal translation (approximately 22 mm).

Despite the lack of a relation between absolute amount of misalignment and peak forces/torques, when taking the direction of misalignment into account a relation did become evident. Regression analysis of the peak forces and torques (Table 5) revealed regression with R-squared larger than 0.7 in Fx ($F{x}_{peak}=-0.04 Alignmen{t}_{transPD}^{2}+0.81 Alignmen{t}_{transPD}-98.83; {R}^{2}=0.75;p<0.01$) and Fz ($F{z}_{peak}=-1.71 Alignmen{t}_{transPD}-10.8; {R}^{2}=0.76;p<0.01$).

**Table 5:** Peak forces and torques presented as M (SD) per proximal/distal translational misalignment trial

Translational alignment proximal/distal [mm]	Fx_peak_ [N]	Fy_peak_ [N]	Fz_peak_ [N]	Mx_peak_ [Nm]	My_peak_ [Nm]	Mz_peak_ [Nm]
− 22.6	− 136.5 (7.3)	2.3 (1.0)	21.6 (2.2)	0.24 (0.01)	0.24 (0.03)	− 0.2 (0.04)
− 22.1	− 138.1 (4.5)	2.1 (0.8)	20.9 (2.1)	0.06 (0.02)	0.22 (0.02)	− 0.18 (0.02)
− 11.8	− 122.7 (10.2)	5.4 (1.8)	19.4 (2.2)	0.44 (0.12)	0.35 (0.05)	− 0.28 (0.07)
− 11.7	− 116.6 (7.1)	8.7 (2.4)	20.0 (2.1)	0.51 (0.03)	0.3 (0.02)	− 0.29 (0.06)
− 4.6	− 103.1 (5.6)	− 1.4 (1.0)	− 5.4 (2.1)	0.08 (0.01)	− 0.09 (0.06)	− 0.15 (0.05)
− 4.0	− 102.6 (4.9)	− 1.0 (0.6)	− 7.2 (0.9)	0.06 (0.02)	− 0.11 (0.07)	− 0.2 (0.05)
− 3.6	− 105.4 (8.3)	− 3.5 (1.2)	− 2.3 (1.2)	0.11 (0.05)	0.04 (0.03)	− 0.12 (0.06)
− 3.2	− 97.3 (15.8)	− 1.2 (3.8)	0.5 (1.9)	0.23 (0.07)	0.07 (0.03)	− 0.16 (0.08)
3.6	− 92.9 (4.7)	− 2.4 (0.6)	− 22.2 (2.1)	0.03 (0.02)	− 0.19 (0.01)	− 0.32 (0.09)
4.8	− 89.8 (7.3)	− 2.3 (1.0)	− 25.1 (1.9)	0.05 (0.02)	− 0.02 (0.13)	− 0.26 (0.12)
11.1	− 95.7 (3.6)	2.4 (0.5)	− 50.2 (2.0)	0.28 (0.03)	− 0.29 (0.04)	− 0.39 (0.07)
12.1	− 100.7 (5.8)	2.0 (0.8)	− 11.0 (1.8)	0.28 (0.02)	− 0.19 (0.14)	− 0.36 (0.09)

### Change of misalignment during flexion

**Fig. 7:**
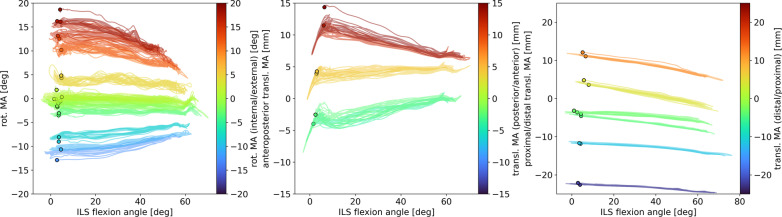
Behavior of misalignment over flexion/extension cycles of each trial. The amounts of misalignment (rot. / transl. MA) are represented by the graph colors and the starting value of each misalignment setting is marked with a circle. Green to blue shades represent internal rotation, posterior translation and distal translation respectively (left to right), while orange–red shades represent external rotation, anterior translation and proximal translation respectively

To assess whether and how the amount of misalignment changes in the dynamic situation during flexion and extension of the ILS and orthosis setup, this behavior is shown in Fig. 7 for all three types of misalignment. The amount of misalignment is lower at maximum flexion than at maximum extension. In proximal/distal translational misalignment, there is little change among the repetitions of the flexion/extension cycle and some trials show a more distal position of the ILS joint at maximum flexion. In addition to that, especially in the stronger rotational misalignment settings and in the strongest anterior translational misalignment setting, a reduction of the misalignment in the first flexion/extension cycle can be observed.

### Effects of misalignment on manually applied flexion torque and maximum flexion angle

**Fig. 8:**
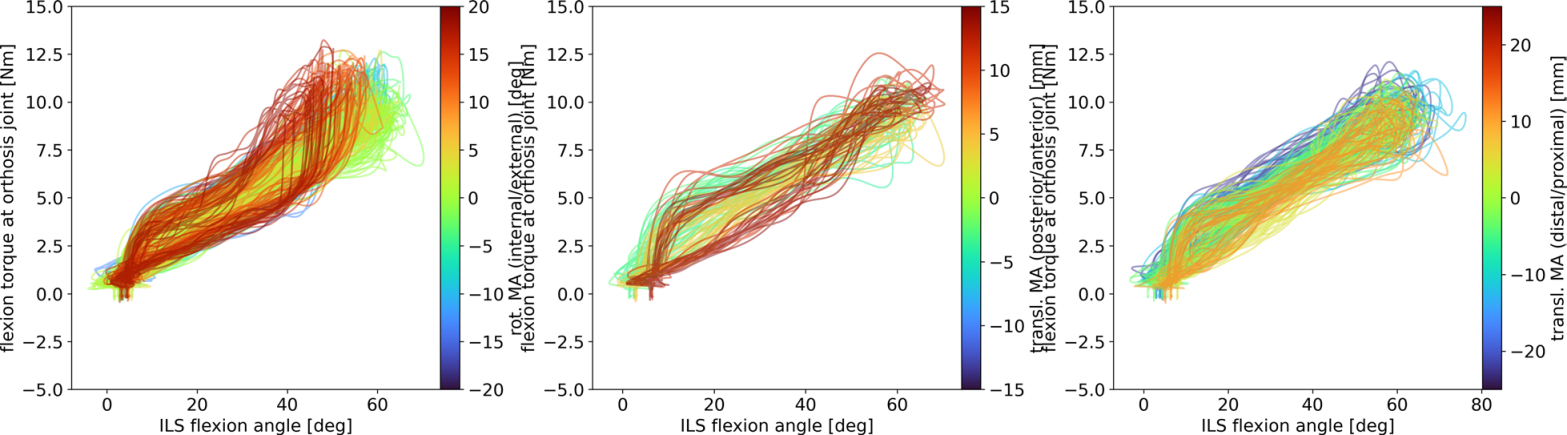
Behavior of flexion torque over flexion/extension cycles of each trial. The amounts of misalignment (rot. / transl. MA) are represented by the graph colors. Green to blue shades represent internal rotation, posterior translation and distal translation respectively (left to right), while orange–red shades represent external rotation, anterior translation and proximal translation respectively

The administered flexion torque at the orthosis joint over the ILS flexion angle in all misalignment settings is shown in Fig. 8. In the translational misalignment settings, there is no visible effect of misalignment on the required flexion torque. The rotational misalignment plot shows a tendency for increased maximum torque and/or a decreased maximum flexion angle in settings with larger misalignments. Regression analysis revealed no relationship between amount of misalignment and peak torque, but did reveal a relation of decreased maximum flexion angle with increased rotational misalignment, which however does not meet the criterium of R^2^ > 0.7 for strong relationships set for this study ($LegFlexionAngl{e}_{peak}=-0.04 Alignmen{t}_{rot}^{2}-0.07 Alignmen{t}_{rot}+60.68; {R}^{2}=0.62;p<0.01$).

## Discussion

In this article, we mimicked the use of an exoskeleton and analyzed effects of different amounts and directions of misalignment using an ILS and a passive knee brace that was manually actuated, revealing an increasing load on the joint with increasing misalignment, supporting our hypothesis. More specifically, an increase in rotational misalignment around the vertical axis resulted in higher absolute forces in mediolateral (y-) direction as well as higher absolute torques around all three axes. The statistical analysis of translational misalignments and resulting joint forces and torques gave different results when testing only absolute values than the analysis of values considering the directions of misalignment and load. This is an indication that the effects on joint forces and loads are not symmetrical in translational misalignments, as opposed to rotational misalignments where both analyses resulted in the same relationships. Considering only absolute values, increased anteroposterior translational misalignment leads to increased absolute peak forces in anteroposterior direction and increased absolute peak ab-/adduction torques. Considering the directions of misalignment and load, an increase in translational misalignment in anteroposterior direction resulted in higher absolute peak forces in mediolateral direction as well as higher ab-/adduction and flexion/extension torques. An increase in translational misalignment in proximal/distal direction resulted in higher absolute forces in anteroposterior and vertical direction when considering directions of misalignment and forces/torques, even though no relationships were present regarding only the absolute values.

To the best of our knowledge, this is the first study showing the relative effect of different directions and amounts of misalignment on joint loads. Previous research using similar knee braces found increased forces in the brace hinge [14] but no significant differences in kinematics [15] with translational misalignment. In those studies, the braces were worn by human subjects and not actuated through any external force application. A cadaver study found that while the well-aligned brace reduced the strain on the anterior cruciate ligament, the misaligned situation showed an increased strain compared to the unbraced knee [16]. Despite the difference in research question and approach, with the present study using this passive knee brace to simulate a powered exoskeleton, the findings of the cadaver study are along similar lines as our outcome of an increasing joint load with increasing misalignment.

Among the rotational misalignment trials, the mean absolute peak force in anteroposterior (x-) direction was highest in a trial with 16.1 deg external rotation of the ILS joint and was 0.7 times or 90.5 N higher than the mean absolute peak forces at close to optimal alignment (0.1 deg internal rotation). The same trial resulted in the highest mean absolute peak torques in all three directions with peak Mx (ab-/adduction) being 3.8 Nm or 377 times higher, peak My (flexion/extension) being 4.44 Nm or 44 times higher and peak Mz (in-/external rotation) being 3 Nm or 12.5 times higher than in the aligned trial. The mean absolute peak force in mediolateral (y-) direction was highest in a trial with the strongest internal rotation (12.9 deg) and was 415 times or 41.5 N higher than in the aligned setting. None of the highest peak forces or torques recorded were associated with the trial with strongest external rotation (18.6 deg). While this is counterintuitive, it might be related to the reduction of misalignment within the first flexion of that trial leading to a lower effective misalignment comparable to the following trial which was the trial with the highest peak values in Fx, Mx, My and Mz. Remarkably, the lowest mean absolute peak force in anteroposterior (x-)direction was observed in a trial with 1.8 deg external rotation and not at the most accurately aligned trial (M (SD) of 77.1 N (12.1 N), which was approximately 20 N higher than at best rotational alignment). This might be due to the fact that the string used for applying flexion torque to the orthosis ripped at the end of the previous trial, which in turn led to a change in anteroposterior alignment (0.7 mm posterior translation while the mean across all rotational misalignment trials was 3.5 mm (SD 1.3 mm) anterior translation. Moreover, the maximum absolute mean peak forces and torques were not observed at the trials with the strongest misalignments, which might indicate that there is a saturation of maximum forces and torques at very large misalignments. This could be confirmed by testing a larger range of misalignments.

Concerning anteroposterior translational misalignment, the ranges of observed mean peak forces and torques were considerably lower than those observed in rotational misalignment. Similarly, the observed differences in mean absolute peak forces in the transversal plane (x- and y-direction) were lower than those observed in rotational misalignment. Both peak absolute Fx and peak absolute Fy were highest in a trial with 11.5 mm anterior translation of the ILS joint and lowest at 3.92 mm anterior translation with a difference of 41.8 N and 4.6 N respectively (being 0.4 times and 0.8 times respectively higher than the lowest observed mean absolute peak forces). The maximum and minimum mean absolute peak ab-/adduction and flexion/extension torques only varied by 0.64 Nm and 0.24 Nm respectively.

In the proximal/distal translational misalignment, the ranges of forces and torques were comparable to those in anteroposterior translational misalignment. The observed differences in maximum and minimum mean absolute peak forces in anteroposterior (x-) direction were also comparable to those observed in the anteroposterior translational misalignment trials with 48.3 N (0.5 times higher at 22.1 mm distal translation than the lowest values, observed at 4.8 mm proximal translation). Mean absolute peak forces in z-direction varied by 49.7 N, the result at 11.1 mm proximal translation being 111 times higher than that at 3.1 mm distal translation.

Overall, we observed much higher peak forces in mediolateral direction, as well as torques in all three planes in the rotational misalignment settings than in the translational misalignment settings. Although it is impossible to relate a certain amount of rotational misalignment to translational misalignment, we observed that rotational misalignments of approximately 10 deg resulted in much higher peak forces in mediolateral direction and peak torques in all planes than translational misalignments of 10 mm in any direction. Peak forces in anteroposterior direction showed similar increases in all types of misalignments. Further, the R^2^-values, specifically in regression of rotational misalignment versus joint forces and torques were very high with values equal to or larger than 0.9, indicating that a large portion of the variation in ILS joint force and torque results can be explained by differences in misalignment. While clinicians anecdotally report proximal/distal translational misalignment as being the biggest problem in practice when using lower limb exoskeletons, the results of this study show that rotational misalignments can also cause a substantial increase in forces and torques and that those effects should not be neglected. As such rotational misalignments can be caused in practice by unprecise positioning of the exoskeleton, limited DOF compared to human motion, or in patients with rotational deformities, their effects should be considered in the risk management process during exoskeleton development, in addition to the perhaps more obvious translational misalignments. The impact of both translational and rotational misalignments depends on the mechanics of the exoskeleton under consideration as compliant parts can alleviate effects of misalignments [5].

We found a reduction in maximum flexion with unchanged flexion torque in stronger rotational misalignments. Furthermore, the amount of misalignment showed a tendency to reduce with increasing flexion during the movement in rotational and anteroposterior misalignments. Since the applied flexion torque as well as forces and torques in the ILS joint increased with increasing flexion, it is indicated that the setup of ILS and orthosis was forced into a more aligned position. This can be attributed to (1) the compliance of orthosis frame and cuffs; (2) the compliance of the soft tissue; and/or (3) slipping of the orthosis cuffs. While this reduction of misalignment was reversed for the most part during extension, a small amount of misalignment reduction occurring in the first flexion/extension cycle was irreversible, specifically with large misalignments. This indicates that at least some of the misalignment reduction can be attributed to slipping or unreversed deformation of soft tissue, which can cause soft tissue injuries upon prolonged or repeated use. In future research, the behavior of the amount of misalignment over time could be further analyzed as it might be an additional indicator for increased load caused by increased misalignment. The exact effects of misalignments at a soft tissue level were not the focus of this study. They should however be investigated in future studies to increase the understanding of misalignments as a hazard in exoskeleton use. Furthermore, the observed levels of forces and torques should be related to comfort and safety of the user, providing robot developers with important knowledge on risks and potential mitigation strategies.

There are some limitations to the setup used in this study. The weight of the ILS (4.2 kg) is clearly lower than a human leg’s weight, which is approximately 16% of the total body weight [17]. However, for the force and torque measurements during swing, only the weight of the lower leg segment is of relevance which, due to the circular weight at its distal end, accounts for most of the ILS weight with 3.3 kg. This would approximately correspond to the weight of the shank and foot of a person weighing 57 kg, although the weight distribution is not realistic as the major part of the weight is placed at the distal end of the ILS [17]. The weight in connection to the procedure used for adjusting the alignment might have caused additional limitations. Force in proximal/distal direction did not show clear trends linked to rotational or anteroposterior translational misalignments and showed a remarkable variation in starting values of each trial. Those starting values might have been influenced by a pre-tensioning effect caused by the amount of force with which the lower leg was pushed upwards by the experimenter during performing misalignment adjustments. A higher or lower starting value might further influence the analyzed peak values of Fz. These effects could be reduced in future experiments by trying to support the lower ILS weight in the same way during each adjustment. Moreover, the upper end of the upper leg rod and the upper orthosis segment were fixed to mimic a situation in which both the subject’s hip joint and the upper orthosis segment remain in one position and to avoid slipping down of the ILS. In a realistic situation however, the exoskeleton and its wearer would either move through the room or the exoskeleton would be fixed at hip level, such as in a stationary robotic gait trainer, which would enable the human leg segments and the knee joint to move in the room. Weight effects are therefore not accurately reconstructed. In addition to that, we are simulating the situation during swing and are not considering a weight bearing situation, which would increase the forces on the musculoskeletal system considerably, especially during sit-to-stand movements [18]. This is supported by a report of a tibia fracture in exoskeleton use which occurred during sit-to-stand motion after an unexpected shutdown that likely caused a misalignment [10]. Those limitations however had to be accepted for the sake of practical feasibility of the setup.

Another aspect of the ILS, which is only a very limited and simple simulation of a real leg, is the soft tissue. We used a polyether foam rubber cylinder SG 40 which mimics the softness and friction of human tissue to some extent but we did not perform any tests to assess how well the real situation can be approached using this material. Also the cylindric shape is a very simplistic approach of recreating the shape of a human leg, however often used as a representation of human body segments, including in technical standards [19]. The effect of the soft tissue characteristics on the outcomes is unknown. However, the reduction of misalignment magnitude during ILS flexion suggests some soft tissue compliance effects and the hysteresis observed in loads might be a sign for soft tissue compression [20]. It would therefore be interesting to explore other approaches for mimicking soft tissue, such as those suggested in literature and standardization [21–23], to better understand the effect of different soft tissue characteristics. In addition to that, the joint that was used for simulating the human knee is a simple hinge with a centered axis of rotation, which does not reproduce the translation of the axis of rotation or the tibiofemoral rotation characterizing an anatomical knee joint [7, 24]. Micro-misalignments caused by the kinematic mismatch between anatomical joint and exoskeleton joint can therefore not be assessed using the current setup. For example, the external rotation of the tibia with respect to the femur accompanying the final 20 degrees of extension in a human knee [24] would lead to a more pronounced misalignment with an external rotational misalignment and thereby to stronger effects on the load. In the present study, the load increase within the first 20 degrees of flexion was low. However, in a weight bearing situation those effects might be more pronounced. Furthermore, the current setup does not allow quantifying the strap pressure which might in theory have an effect on the behavior and resulting forces and torques. Specifically in translational misalignment trials, where the straps had to be re-attached after each adjustment, the varying and non-standardized strap pressures could be a concern. However, a comparison of rotational misalignment effects with very tight and loosened straps resulted in no clear differences. Assuming that the effect of varying strap pressures would also be negligible in other misalignment conditions, we can have increased confidence that the required re-attachment of straps did not affect the results in the translational misalignment trials. Therefore, despite the discussed limitations of the set-up itself, these factors should not affect analysis of the differences between different misalignments. It does, however, hinder generalization of the results to real-life situations in terms of absolute forces/torques.

Other limitations are connected to the use of a passive orthosis instead of an active exoskeleton. The orthosis used in this study is a passive knee brace commonly used for providing stability after knee joint ligament injuries. Its exact kinematics and stiffness might differ from the behavior of exoskeletons which affects the generalizability of the results. The same amounts and directions of misalignments might lead to different changes in joint load when other devices are considered, due to variations in joint kinematics and compliance [5]. As each exoskeleton design and mechanical properties vary per device, a degree of uncertainty will remain. We replaced the actuation of an exoskeleton with a pulling force exerted on the lower orthosis segment by an experimenter. This is a simple and effective method of mimicking exoskeleton actuation but it underlies some uncertainties and variations. We observed a reduced maximum flexion with increased rotational misalignment against unchanged maximum flexion torque at the orthosis joint. The same flexion torque was therefore reached at lower flexion angles with higher misalignments. The experimenter likely felt the resistance and did not pull harder to reach the same flexion angle but instead kept the maximum pulling force relatively constant. Forcing the same flexion angle in all conditions would better mimic the behavior of an exoskeleton with trajectory control and might have led to higher absolute peak forces and torques. A standardization of the peak flexion angle using an electro-goniometer or a static indicator for the desired lower leg position could be considered for future research. However, such an approach might cause breakage of certain setup parts and the current approach, while more subjective and prone to variation, might mimic the torque control of an actuated exoskeleton. Another limitation is connected to the calculation of misalignment magnitudes. As the resting state before the first flexion was used to obtain the misalignment values per trial and misalignment was often irreversibly reduced during the first flexion/extension cycle, the amount of misalignment might better be calculated based on the situation after a few flexions or at the end of a trial. It is however relevant to note that this reduction in misalignment takes place, suggesting that injuries might occur in the very first step as either the biological structures are forced to move in unnatural directions or the cuffs of the exoskeleton slip on the skin. In addition to that, we noticed slight misalignments in the directions not targeted for misalignment. For example, when the anteroposterior position of the ILS joint was not supposed to be misaligned and was intended to be kept at 0 mm, it did show an anterior translation of 4.8 mm on average with a standard deviation of 2.6 mm. Those small misalignments in other directions than the direction analyzed in the respective trials can affect the outcomes. They were however small compared to the target misalignments and a perfect alignment is unlikely to be achieved in any exoskeleton use situation.

Those limitations in the setup and procedure might reduce the reliability of the absolute values of joint forces and torques measured in this setup, as an anatomical leg might behave differently and damping of forces and torques through soft tissue might not be perfectly mimicked. A potential solution is to improve the ILS setup by adding a different type of soft tissue, which has documented characteristics resembling human soft tissue [19, 25]. However, despite these limitations, the current study does show the relative effect of different amounts of misalignment on joint forces and torques, supporting the hypothesis that translational and rotational misalignments increase the load on the knee joint.

Despite these limitations, present findings are a first step towards understanding the effects of misalignments on the musculoskeletal system. We showed that misalignments of a lower leg exoskeleton can lead to a manifold increase of internal knee forces and torques compared to those experienced in a well-aligned situation. This is supporting the need for carefully considering hazards associated with not only translational but also rotational misalignments during wearable robot development and use. To support robot developers in validation of this and other safety aspects, the European project COVR provides procedures for validation tests in an online Toolkit (www.safearoundrobots.com) [26, 27]. Future research can increase this knowledge by investigating more conditions and exploring options to develop improved limb simulators. A setup that can measure effects of misalignments in a weight bearing situation would increase the impact of the results. Furthermore, the knowledge base should be extended by investigating effects of misalignment on soft tissue level and by relating the observed effects to comfort and safety in real human subjects.

## Conclusion

The present work assessed the effects of different amounts of rotational and translational misalignments during exoskeleton use on musculoskeletal forces using a novel ILS and a manually actuated orthosis. To our knowledge this is the first study showing the relative effect of different amounts and directions of misalignment on joint forces and torques. We found that knee joint forces and torques increase in various directions when a misalignment is introduced. In general, we saw an increasing load on the knee joint with increasing misalignment. Specifically, larger internal rotation of the ILS knee joint with respect to the orthosis joint led to higher forces in medial direction in the knee joint as well as higher adduction, flexion and external rotation torques. Stronger external rotation of the ILS knee joint with respect to the orthosis joint led in turn to higher lateral joint forces as well as higher abduction, flexion and internal rotation torques. Stronger posterior translation of the ILS joint with respect to the orthosis joint led to higher lateral knee forces as well as higher abduction and flexion torques while stronger anterior translation of the ILS joint with respect to the orthosis joint led to higher medial knee forces as well as higher abduction and extension torques. Stronger distal translation of the ILS joint with respect to the orthosis joint led to higher posterior and compression forces while proximal translation of the ILS joint tended to lead to increased posterior and tensile forces. These findings show that all types of misalignment, including rotational misalignment, should be considered specifically when designing and applying exoskeletons for rehabilitation, assistance or augmentation of human functioning. Future research should focus on improved dummy limbs, testing with actuated exoskeletons and testing the weight bearing situation, as well as on the potential effects of increased loads, to create a knowledge base on the effects of exoskeleton misalignments.

## Data Availability

The datasets used and/or analyzed during the current study are available from the corresponding author on reasonable request.

## Annex

See Table 6.

**Table 6:** Description of used marker set

Marker name	Placement description
FTx	FT sensor on top rim front (marks positive x-direction)
FTConn*	FT sensor on top of connector block
FTBlat	FT sensor on top rim lateral-back side (relative to orthosis lateral/medial definition)
FTBack	FT sensor on top rim mid back
FTFL*	FT sensor on top rim front lateral (relative to orthosis lateral/medial definition)
FTFM	FT sensor on top rim front medial (relative to orthosis lateral/medial definition)
AxisLat	Lateral point physical axis leg joint
AxisMed	Medial point physical axis leg joint
ULTop*	Top marker on upper leg—frame mounting connector
ULFront	Front marker on upper leg—frame mounting connector
ULBack	Back marker on upper leg—frame mounting connector
LowerLegBack*	Marker at mid back of circular weight
LowerLegLat	Marker at most lateral position of circular weight
LowerLegMed	Marker at most medial position of circular weight
XULLFrame	Orthosis UpperLeg lateral location on frame fixation point
XULMFrame	Orthosis UpperLeg medial location on frame fixation point
XULM1*	Upper marker placed on medial upper orthosis frame
XULM2	Middle marker placed on medial upper orthosis frame
XULMJoint	Marker at the upper part of the medial joint plate of the orthosis
XULL1*	Upper marker placed on lateral orthosis frame
XULL2	Middle marker placed on lateral orthosis frame
XULLJoint	Marker at the upper part of the lateral joint plate of the orthosis
XLLMJoint	Marker at the lower part of the medial joint plate of the orthosis
XLLM1*	Middle marker placed on medial lower orthosis frame
XLLM2	Lower marker placed on medial lower orthosis frame
XLLLJoint	Marker at the lower part of the lateral joint plate of the orthosis
XLLL1*	Middle marker placed on lateral lower orthosis frame
XLLL2	Lower marker placed on lateral lower orthosis frame
XLLRopeAttachment	Mid back marker on lower strap of lower leg part orthosis, where rope is attached
1D_ForceSensor1	Proximal (to leg) top point on sensor
1D_ForceSensor2	Distal top point on sensor
1D_ForceSensor3	Proximal bottom point on sensor
1D_ForceSensor4	Distal bottom point on sensor

Markers marked with an asterisk were not used for the calculations in this article but were placed for potential reconstruction of missing markers and further analysis. Markers FTFM, FTBack, FTBLat and FTx define the FT sensor segment, AxisMed, AxisLat, ULFront and ULBack define the upper ILS segment, AxisMed, AxisLat, LowerLegMed and LowerLegLat define the lower ILS segment, XULMJoint, XULLJoint, XULM2 and XULL2 define the upper orthosis segment, XULMJoint, XULLJoint, XLLM2 and XLLL2 define the lower orthosis segment, and XLLRopeAttachment, 1D_ForceSensor1, 1D_ForceSensor2, 1D_ForceSensor3 and 1D_ForceSensor4 define the tensile force sensor segment

## Abbreviations

DOF: Degrees of freedom
FT: Force and torque
ILS: Instrumented leg simulator
SG: Specific gravity
